# Nudging General Practitioners to explore suicidal thoughts among depressed patients

**DOI:** 10.1186/s12875-023-02043-3

**Published:** 2023-04-01

**Authors:** Elke Elzinga, Derek P. de Beurs, Aartjan T.F. Beekman, Otto R. Maarsingh, Renske Gilissen

**Affiliations:** 1 113 Suicide prevention, research department, Paasheuvelweg 25, Amsterdam, 1105 BP the Netherlands; 2 Amsterdam Public Health research institute, Amsterdam UMC, Vrije Universiteit Amsterdam, De Boelelaan 1117, Amsterdam, The Netherlands; 3 Department of Clinical Psychology, University of Amsterdam, Amsterdam, The Netherlands; 4 Department of Psychiatry, Amsterdam UMC, Vrije Universiteit, Amsterdam, The Netherlands; 5 Department of Research & Innovation, GGZ inGeest Specialized Mental Health Care, Amsterdam, The Netherlands; 6 Department of General Practice, Amsterdam UMC, Amsterdam Public Health Research Institute, Amsterdam, The Netherlands

**Keywords:** Suicide prevention, Suicide exploration, General practitioners, General practice, Mental health, Depression, Intervention, Nudging, Behaviour change, Clinical guidelines.

## Abstract

**Background:**

While frank discussion of suicidal thoughts in patients with depression is important for the prevention of suicide, suicide exploration of General Practitioners (GPs) is suboptimal. This study aimed to assess whether an intervention that prompts pop-up screens nudges GPs to more frequently explore suicidal thoughts over the course of two years.

**Methods:**

From January 2017 to December 2018, the intervention was incorporated in the information system of the Dutch general practice sentinel network. New registration of an episode of depression triggered a pop-up screen referring to a questionnaire about GPs’ behaviour with regard to exploring suicidal thoughts. In two years, 625 questionnaires were completed by GPs and analysed using multilevel logistic regression analyses.

**Results:**

Compared to the first year, GPs were 50% more likely to explore suicidal thoughts among patients in the second year (OR 1.48; 95%CI 1.01–2.16). When adjusting for patients’ gender and age we found that the effect of the pop-up screens disappeared (OR 1.33; 95% CI 0.90–1.97). Suicide exploration occurred less frequently in women than in men (OR 0.64; 95% CI 0.43–0.98) and in older compared to younger patients (OR 0.97; 95% CI 0.96–0.98 per year older). In addition, 26% of variation in suicide exploration was because of differences in general practice. There was no evidence that general practices developed differently over time.

**Conclusions:**

Although low cost and easy to administer, the pop-up system was not effective in nudging GPs to explore suicidality more frequently. We encourage studies to test whether implementing these nudges as part of a multifaceted approach will lead to a stronger effect. Moreover, we recommend researchers to include more variables, such as work experience or previous mental health training, to better understand the effects of the intervention on GPs’ behaviour.

## Background

Worldwide, over 700.000 people die every year as a result of suicide and approximately 20 times as many attempt suicide [1, 2]. In addition, an estimated 8% of people experience suicidal thoughts at some point in their live [3]. Suicidal thoughts are an important risk factor for suicidal acts; over a quarter of people who experiences these thoughts attempts suicide later in life [4]. Early recognition and intervention of patients at risk for suicide is therefore crucial.

In countries with a primary care network, such as the Netherlands, General practitioners (GPs) are the core of the health care system. They function as gatekeepers in referring patients to specialized care and often have enduring relationships with patients [5]. Therefore, GPs have a large exposure to patients who are potentially at risk for suicide. In fact, people who died by suicide were more often in contact with a GP than with mental health services prior to death [6]. A Dutch study reported that in the month before their suicide or attempted suicide, about half of the patients had consulted a GP [7]. Supporting primary care is therefore seen as one of the most effective elements of suicide prevention strategies [8–10].

In primary care, suicide prevention practices are mostly delivered by physicians, and their behaviour serves as proxy for quality of care. Improving the quality of care often requires physicians to change behaviour in accordance with evidence-based practice or clinical guidelines [11]. Various clinical guidelines recommend assessing the presence of suicidal thoughts among patients with an elevated risk, such as patients diagnosed with depression [12, 13]. Regardless, in a previous study we found that assessment of suicidal thoughts occurs in about two third of depression-related consultations [14]. Other studies have also shown that suicide exploration rates in primary care are suboptimal [15–17].

Many strategies have been developed to influence professional behaviour, although most consist of providing education or training [18, 19]. Suicide prevention interventions in primary care also mostly rely on the provision of education and training [20]. Although widely recommended, most studies were only able to report short-term effects on confidence, knowledge or skills, and fail to report long-term effects on behaviour change [21, 22]. Changing behaviour is complex and often requires more than influencing knowledge or skills [19]. An important overview study of systematic reviews on changing provider behaviour also emphasized that education provides small and short-term effects. More passive methods, such as the delivery of information and materials, are only effective for creating awareness. Issuing reminders was the most promising individual intervention to substantially change medical practice, especially for the provision of preventive care [23]. Besides, developing a reminder system is a relatively inexpensive intervention that is easy to implement [24]. Issuing reminders may thus be a potential strategy to change provider behaviour and increase suicide exploration rates in general practice. To our knowledge, this has not been tested before.

This study aims to assess whether a pop-up reminder system nudges GPs to more frequently explore suicidal thoughts over the course of two years. We implemented a pop-up intervention in GPs’ Electronic Health Record (EHR) to alert GPs to explore suicide risk in patients consulting for depression. Because of the great variability in suicide exploration of GPs we found in a previous study [14], we also tested if general practices, as hypothesized, show a different development over time.

## Methods

### Design and setting

This pre-post study was performed among the network of Dutch Sentinel general practices of the Nivel Primary Care database [25]. The sentinel network consists of approximately 40 general practices, which provide in-depth health care information on illnesses and procedures that cannot be obtained from electronic medical records. In 2018, the network of sentinel general practices had almost 130.000 registered patients and was representative in terms of patients’ age, gender, geographical distribution, and population density [26].

We created an automated pop-up screen that was implemented in GPs’ EHR. The pop-up screen was activated after recording a new episode of depression during a consultation. This pop-up screen referred to a questionnaire designed to gather data about GPs’ suicide exploration behaviour, described previously [14]. Within this infrastructure, it was not possible to create a control group because the pop-up functioned as both intervention and measurement instrument. Effect of the pop-up screen was therefore only assessed in relation to consultation year.

### Instrument and measures

The pop-up screen itself included some control questions about the consultation to make sure it concerned (1) a regular patient of the GP, (2) a patient with depression, (3) a new episode of depression, and (4) a face-to-face consultation. If these were answered affirmatively, the follow-up questionnaire was activated. GPs could access the questionnaire straight away or complete it at a later moment. The full questionnaire included 19 items, but not all questions were applicable during all consultations. On average, it took 66 seconds to complete the questionnaire. Our dichotomous main outcome measure ‘suicide exploration’ was assessed with the question: “have you asked the patient if he/she experiences suicidal thoughts?”.

### Data collection

Data was collected from January 2017 until December 2018. General practices that did not provide data over the full study period were excluded, yielding a total of 35 general practices. The present study is based on consultations with patients who were diagnosed with a new episode of depression.

### Analyses

We analysed our data using multilevel logistic regression techniques. The first model only includes year of consultation and was adjusted for clustered data of patients within general practices (two level structure). Since we found in a previous study that suicide exploration occurred more frequently in male and younger patients [14], we created a new model where we adjusted for these variables. The Intraclass Correlation Coefficient (ICC) is a measure to calculate the between group variance as proportion of the total variance and is used to estimate variation between general practices [27]. We added a random slope for consultation year to the model and used the likelihood ratio test to assess whether the general practices showed different development over time. We computed and plotted the random effects to specify the variation between general practices.

The analyses were performed using RStudio Statistical Software (version 2021.09.1) using lme4 and ggeffects packages [26, 27]. The significance level was set at < 0.05.

## Results

Table 1 presents the descriptive statistics per year and in total. In total, we included 625 completed questionnaires about consultations with patients consulting for a new episode of depression. The majority (60%) were women, mean age of the patients was 49.9 years old (sd 19.0). Overall, in 69% of the consultations, suicidal feelings were explored and in 45% of those, patients reported suicidal feelings.

**Table 1:** Descriptive statistics of all variables of the model presented per year and overall

	2017	2018	Total
**Patients with depression**	335	290	625
**Patient gender (%)**			
Male	129 (39%)	120 (41%)	249 (40%)
Female	206 (61%)	170 (59%)	376 (60%)
**Patient mean age (sd)**	51.9 (18.9)	47.5 (18.8)	49.9 (19.0)
**Suicide exploration (%)**			
Yes	220 (66%)	213 (73%)	433 (69%)
No	115 (34%)	76^1^ (27%)	191 (31%)
**Prevalence of suicidal feelings (%)** ^**2**^			
Yes	91 (41%)	102 (48%)	139 (45%)
No	129 (59%)	111 (52%)	240 (55%)

Percentages are presented for the columns

^1^ Does not add up to 290 due to missing n = 1

^2^ Of consultations during which suicidal feelings were explored.

Table 2 shows the results of the multilevel analyses with a random intercept on general practice level. The first model shows the influence of contact year on suicide exploration while adjusted for nested data of consultations within general practices. This model shows that the number of consultations during which suicide was explored was significantly higher in 2018 compared to 2017 (OR 1.48; 95%CI 1.01–2.16). The ICC that was calculated for this model is 0.24, meaning that 24% of the variation in suicide exploration is explained by general practice.

In the second model, which is adjusted for patients’ gender and age, the effect of consultation year on suicide exploration of GPs attenuated (OR 1.33; 95%CI 0.90–1.97). Female gender and older age are both negatively correlated with suicide exploration (respectively OR 0.64; 95% CI 0.43–0.98 and OR 0.97; 95% CI 0.96–0.98 per year older). The ICC for this model is 0.26, indicating that 26% of the variation in suicide exploration among patients is explained by general practices. We also created a model with a random slope for consultation year to assess whether suicide exploration of general practices developed differently over time. According to the log likelihood ratio test, this was not the case (X^2^[2] = 1.212, P = 0.545). This model is therefore not presented here.

**Table 2:** Multilevel logistic models for the effect of consultation year on suicide exploration

	Model 1			Model 2		
Predictors	Odds Ratios	CI	p	Odds Ratios	CI	p
**(Intercept)**	2.33	1.48–3.66	< 0.001	12.72	5.72–28.27	< 0.001
**Consultation year: 2018 (Ref = 2017)**	1.48	1.01–2.16	0.045	1.33	0.90–1.97	0.158
**Patients’ gender: Female (Ref = Male)**				0.64	0.43–0.98	0.027
**Patients’ age**				0.97	0.96–0.98	< 0.001
**Random Effects for general practice**						
**Within-group (residual) variance**^**1**^	3.29			3.29		
**Between-group variance**	1.06			1.13		
**ICC**	0.24			0.26		
**N**	35			35		
**Observations**	624			624		
**Marginal R2 / Conditional R2**	0.009 / 0.250			0.070 / 0.308		

CI = Confidence Interval, ICC = Intraclass Correlation Coefficient

^1^ The within-group (residual) variance in logistic regression is equal to π2/3

**Fig. 1:**
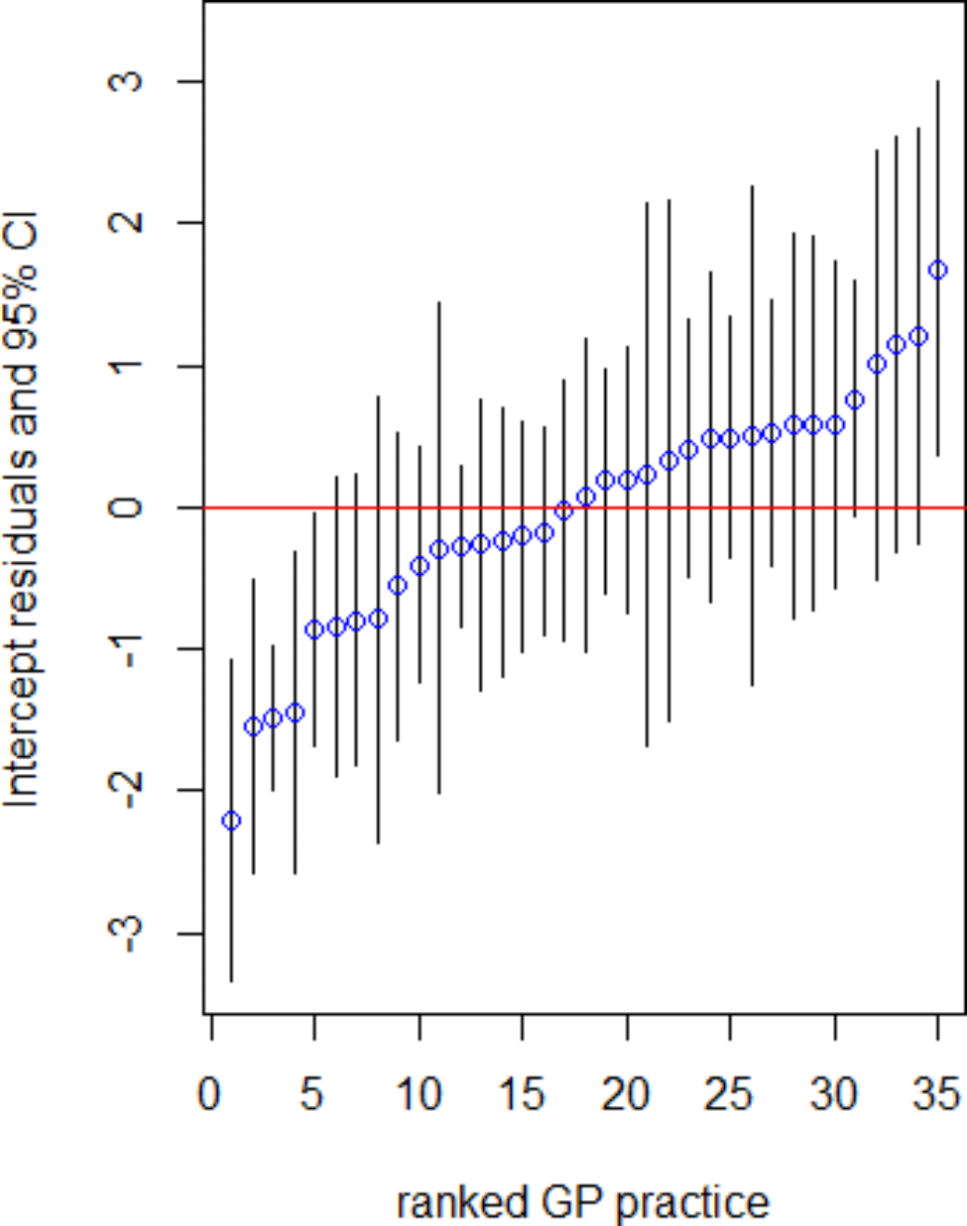
Caterpillar plot of ranked residuals for general practices with 95% confidence intervals for log-odds of exploring suicidal thoughts

In Fig. 1 the random effects (intercepts) of general practices are plotted to show how suicide exploration differs per general practice. The lowest value represents a general practice where suicidality was explored in 1/12 depressed patients and the highest value represents a general practice where this was explored in 17/17 patients.

## Discussion

This study shows that patient-specific pop-up reminders were not effective in nudging GPs to more frequently explore suicidal thoughts over a period of two years. GPs were 50% more likely to explore suicidal thoughts in the second year compared to the first, however, this effect disappeared after adjusting for patients’ gender and age. Though there was considerable practice variation in suicide exploration, general practices showed no different development over time.

Issuing reminders effectively changed professional behaviour in previous studies across a range of settings [23, 24, 28, 29]. Shea at al. reported that issuing reminders was effective for improving preventive services with 77% overall. However, not all preventive services were improved; screening for cervical cancer, for instance, was not. The authors speculate this is related to contextual factors, specifically the time it takes to perform a pelvic examination and patients’ resistance for this procedure [30]. The reason why we did not find evidence for the effectiveness of the reminder system in the present study, may be because of the suicide prevention context, which is perceived difficult and complex by many primary care professionals [31–33]. In order for interventions to effectively change professionals’ behaviour, it is important to adjust them to the context and consider barriers that are in place. For suicide prevention, important barriers are lack of knowledge, skills and/or confidence, lack of time, and limited access to mental health care [31–37].

Most interventions to change suicide prevention practices of professionals include providing education or training [19, 20]. This addresses the individual barriers lack of knowledge, skills and/or confidence. However, in order to effectuate true behaviour change, the environmental barriers should be addressed too. These are much harder to influence because they often require changes on system level. Effective interventions in primary care that address these include the institution of specialized nurses or other health care professionals, organizational changes to increase collaboration within primary care professionals, or collaborative or shared care practices [18]. Not all these interventions have been specifically evaluated for suicide prevention. The mental health support staff was introduced in primary care about a decade ago. This positively influenced suicide prevention practices and was highly valued by GPs [31]. A collaborative care model for suicide prevention was recently implemented and tested. Although we are awaiting the long-term results, short term results are promising and stakeholders especially valued the chain of care element to support collaboration [38].

The Behaviour Change Wheel (BCW) is a contemporary model for behaviour change that can be used for understanding and influencing behaviour in context. In the core of the wheel is the COM-B system, containing the three constructs of behaviour (B): Capability, Opportunity and Motivation. Surrounding the core are nine intervention functions, such as education, training, enablement, and environmental restructuring. These can be used to address deficits in one or more of these behaviour constructs. Education and training influence professionals’ *capability* and *reflective motivation*. Providing pop-ups in the EHR system to remind clinicians to engage in a certain behaviour is a type of environmental restructuring. Prompting these questions restructures the physical context, and influences the *opportunity* and *automatic motivation* conditions of clinicians’ behaviour [39]. This increases the chance that newly adopted behaviour is structurally applied. Combining these with other interventions to overcome the environmental barriers for suicide prevention in primary care modifies behaviour more sustainably. This is in line with recommendations from other studies, arguing that multifaceted interventions targeting various barriers and influencing multiple behaviour constructs simultaneously are more likely to effectuate behaviour change and improve patient-level outcomes [18, 19, 23].

### Strengths & limitations

The naturalistic design of this study was a major strength. We developed a feature that was inexpensive and easy to incorporate in the GPs’ natural workflow. Especially for GPs this is important, because they are known to have restricted time, so developing interventions that add to their high workload will only increase their burden further. The fact that we collected over 600 completed questionnaires in two years shows we were able to adapt to their workflow and create engagement among these GPs.

However, the design also caused some important limitations. First, it was not possible to compare the effects of the intervention group with a control group. Therefore, we cannot rule out whether any of the results are influenced by other extraneous variables. Second, only a limited number of variables were available for this study. Variables we expected to be of influence, such as work experience or previous mental health training [40], are missing. This limited our possibilities to adjust for confounding or effect modification. Finally, implementing pop-up reminders in the EHR system has become more prevalent in clinical practice. Even to the point that it may result in ‘alert fatigue’, causing health care professionals to ignore alerts due to the overload of prompts and reminders [41]. Unfortunately, this data was not available so we could not take it into consideration.

We recommend researchers to test whether implementing these nudges as part of a multifaceted approach will lead to a stronger effect on GPs’ suicide exploration. Further, we recommend adopting a design that allows for comparison with a control group and to include more GP- en general practice variables to develop better understanding of the effect of the reminder system. Previous studies among the sentinel practices have indicated some clinical factors that are associated with suicide attempts and suicides, such as high consultation frequency and other psychological complaints or disorders [42]. Furthermore, suicidal thoughts should also be assessed during later consultations concerning depression, and, preferably, during consultations concerning depressive mood or other psychological complaints. Future studies are therefore recommended to broaden their scope and include more illnesses and complaints. Finally, these insights and insights about sociodemographic variables should be used to improve the accuracy of the reminders and determine whether this affects the effectiveness of the pop-up system.

## Conclusions

Developing a pop-up intervention and implementing it in the GPs’ information system is a low-cost and easy to administer intervention. Unfortunately, we were not able to report a robust effect of the intervention on GPs’ suicide exploration behaviour. We encourage studies to test whether implementing these nudges as part of a multifaceted approach to improve suicide prevention practices of GPs is more effective. In addition, we recommend researchers to include more GP- and general practice variables to better understand the effect of the intervention on GPs’ behaviour.

## Data Availability

The datasets used during the current study are available from the corresponding author upon reasonable request.

## Abbreviations

BCW: Behavioural Change Wheel
CI: Confidence Interval
EHR: Electronic Health Record
GP: General practitioner
ICC: Intraclass correlation coefficient
OR: Odds Ratio
sd: Standard Deviation.
